# Altered Blood Levels of Anti-Gal Antibodies in Alzheimer’s Disease: A New Clue to Pathogenesis?

**DOI:** 10.3390/life11060538

**Published:** 2021-06-09

**Authors:** Antonella Angiolillo, Alessandro Gandaglia, Alessia Arcaro, Andrea Carpi, Fabrizio Gentile, Filippo Naso, Alfonso Di Costanzo

**Affiliations:** 1 Centre for Research and Training in Medicine of Aging, Department of Medicine and Health Sciences “V.Tiberio”, University of Molise, Località Tappino, 86100 Campobasso, Italy; alessia.arcaro@unimol.it (A.A.); gentilefabrizio@unimol.it (F.G.); alfonso.dicostanzo@unimol.it (A.D.C.); 2 Biocompatibility Innovation (BCI), via Lorenzo De Antoni 17/19, Este, 35042 Padova, Italy; alessandro.gandaglia@biocompatibility.bio (A.G.); andrea.carpi@biocompatibility.bio (A.C.); filippo.naso@biocompatibility.bio (F.N.)

**Keywords:** alpha-gal epitope, Alzheimer’s disease, microbiota

## Abstract

Alzheimer’s disease is a neurodegenerative disorder whose pathological mechanisms, despite recent advances, are not fully understood. However, the deposition of beta amyloid -peptide and neuroinflammation, which is probably aggravated by dysbiotic microbiota, seem to play a key role. Anti-Gal are the most abundant xenoreactive natural antibodies. They are supposed to stem from immunization against the gut microbiota and have been implicated in the pathogenesis of several diseases, including multiple sclerosis. These antibodies target the alpha-Gal epitope, expressed on the terminal sugar units of glycoprotein or glycolipid of all mammals except apes, Old World monkeys and humans. The alpha-Gal is constitutively expressed in several bacteria constituting the brain microbiota, and alpha-Gal-like epitopes have been detected in gray matter, amyloid plaque, neurofibrillary tangles and corpora amylacea of the human brain, suggesting a potential link between anti-Gal and Alzheimer’s disease etiopathogenesis. For the first time, our study searched for possible alterations of anti-Gal immunoglobulin levels in Alzheimer’s disease patients. IgG and IgM blood levels were significantly lower, and IgA significantly higher in patients than in healthy subjects. These results suggest that such immunoglobulins might be implicated in Alzheimer’s disease pathogenesis and open new scenarios in the research for new biomarkers and therapeutic strategies.

## 1. Introduction

Alzheimer’s disease (AD), the most common form of dementia, is a progressive, irreversible and incurable neurodegenerative disease, which constitutes one of the major causes of dependency, disability and mortality [1]. There are over 50 million people worldwide living with dementia in 2020. This number will almost double every 20 years, reaching 82 million in 2030 and 152 million in 2050. AD accounts for 50–75% of dementia cases [2].

Despite the huge amount of research, the pathological mechanisms underlying the onset and progression of the disease are not fully understood. Neurodegeneration is most likely caused by an abnormal accumulation of the beta amyloid peptide which produces a cascade of events, including an inflammatory process mainly mediated by microglial activation and formation of neurofibrillary tangles that culminate in neuronal death. The triggering mechanism of this cascade is still unknown and therefore biomarkers with possible mechanistic insights into the AD pathophysiologic processes are urgently needed [3]. Recent works suggest a potential association between gut microbiota, neuroinflammation and AD, either directly owing to bacterial invasion of the brain due to leakage of the blood–brain barrier (BBB) and production of toxins and inflammation, or indirectly by modulating the immune response [4].

The α-Gal epitope (Galα1-3Galβ1-4GlcNAc-R) is a short carbohydrate sequence terminally located in the oligosaccharide unit of glycoproteins and glycolipids, commonly expressed at the cell surface of various microorganisms (protozoa, bacteria, fungi, viruses), marsupials and non-primate mammalians. This epitope is produced by the α1,3-galactosyltransferase (α1,3GT) enzyme [5,6,7,8,9]. In humans, the gene that encodes for this enzyme (α1,3GT or GGTA1 gene) is suppressed and the consequent absence of α-Gal on the surface of cells induces a massive production of anti-Gal antibodies [10]. Anti-Gal IgG, IgM and IgA, in fact, represent over 1% of all circulating immunoglobulins [11]. Their synthesis is thought to be mainly stimulated by chronic exposure to enteric bacteria, various pathogens and environmental antigens presenting the α-Gal epitope on the surface. Such induced immune response represents an evolutionary advantage for the human organism that acquires immune protection against these pathogens [12]. In effect, anti-Gal antibodies fulfil many important roles, such as ensuring immunity from infections originating from several micro-organisms; contributing to rejection in the case of animal-to-human cell, tissue and organ transplantation; accelerating the process of wound healing. However, circulating anti-Gal antibodies have been implicated in the pathogenesis of autoimmune diseases, such as Henoch–Schönlein purpura, IgA nephropathy, rheumatoid arthritis, Crohn’s disease and Graves’ disease [8,9,10,13,14]. Variations in the levels of anti-Gal have also been described in multiple sclerosis (MS) together with an altered microbiome, suggesting a link between anti-Gal antibodies and the disease [15,16]. 

In physiological conditions the anti-Gal antibodies cannot cross the BBB, so they are not present in the cerebrospinal fluid (CSF) of healthy individuals. However, varying titers of anti-Gal Ig were found in the CSF of patients with MS, Guillain-Barré syndrome and meningitis, suggesting that inflammatory processes might alter the BBB and favour the passage of blood Ig (including anti-Gal) into the CSF [17]. Furthermore, α-Gal-like epitopes have been detected in the brain, more precisely in the gray matter of healthy individuals who died accidentally [18], other than in the amyloid plaques, neurofibrillary tangles and corpora amylacea of patients with dementia, including AD [19]. Together with recent literature findings demonstrating the α-Gal epitope presence in several bacteria [20] constituting the brain microbiome [21], these observations suggest a potential relationship between anti-Gal antibodies and AD etiopathogenesis. 

In this study we looked for the first time at possible alterations of anti-Gal Ig levels in AD patients, speculating on the potential role of such antibodies in the pathogenesis of the disease. 

## 2. Materials and Methods

### 2.1. Patients and Healthy Controls

The participants in the study (n. = 60) were consecutively recruited at the Centre for Research and Training in Medicine of Aging of the University of Molise (Italy). The AD patients (n. = 30) fulfilled the National Institute of Aging and Alzheimer’s Association diagnostic criteria for “probable AD with documented decline” [22]. They scored < 24 on the Mini Mental State Examination and >0.5 on the Clinical Dementia Rating. To rule out other potential causes of cognitive impairment, all patients underwent blood tests (including full blood count, erythrocyte sedimentation rate, urea nitrogen and electrolytes, thyroid function, vitamin B12, and folate) and brain imaging. Thirty sex/age-matched cognitively healthy subjects (HS) were recruited as the control group. Since anti-Gal antibody levels can be altered in the context of different pathologies and treatments, subjects with pathologies, such as rheumatoid arthritis, interstitial cystitis, eosinophilic esophagitis [23], Henoch–Schönlein purpura, IgA nephropathy, Crohn’s disease and ulcerative colitis [24], or treated with anticancer such as Cetuximab (or Erbitux) [25], animal derived tissue patches, cartilaginous grafts or bioprostheses such as biological heart valves [26], were excluded. The clinical and demographic characteristics of the two groups of participants are summarized in Table 1.

The study was conducted in accordance with ethical principles stated in the Declaration of Helsinki, and with approved national and international guidelines for human research. The Institutional Review Board of the University of Molise approved the study (Prot. n. 007-08-2018). Written informed consent was obtained from participants or caregivers.

### 2.2. Blood Collection and Processing

The blood collection was performed between 8:00 and 8:30 a.m. after overnight fasting of at least 8–10 h. Venous blood was collected with a vacutainer system (Becton & Dickinson, Milan, Italy). To obtain the serum, the blood was centrifuged within 2 h at 1500× *g* for 10 min. The samples were then stored at −80 °C until their use.

### 2.3. Determination of α-Gal Antibody Titers 

The evaluation of the different human anti α-Gal antibodies isotypes (IgG, IgM and IgA) was performed in duplicate and triplicate using a modified ELISA test (patent EP2626701). Data were expressed as the absorbance value. Each patient’s serum was diluted 50-fold with Phosphate-Buffered Saline (PBS, Merck Lifescience, Darmstadt, Germany) in a final volume of 2 mL. 

Briefly, for each isotype, a Polysorp 96-well plate (Nunc, Rochester, NY, USA) was coated with 100 µL of α-Gal/human serum albumin (HSA) (Dextra Laboratories, Berkshire, UK), 5 µg/mL, for 2 h at 37 °C. After washing three times with PBS, the blocking procedure was performed using 300 µL per well of 2% HSA for 2 h, at RT in darkness. Wells were then washed three times, as above. A set of four wells for each column was loaded with 100 µL of a single diluted serum and the plate incubated for 2 h at 37 °C. After washing, the proper secondary horseradish peroxidase (HRP)-conjugate antibody (1:100) was loaded (anti-human IgG, anti-human IgM and anti-human IgA, Merck Lifescience, Darmsadt, Germany) and the plate incubated for 1 h at 37 °C. After washing, 100 µL of HRP substrate buffer was added to each well for 5 min, at RT, in the darkness. The plate absorbance was measured at 450 nm by a microplate spectrophotometer (Multiskan Sky, Thermo Fisher Scientific, Waltham, MA, USA).

### 2.4. Statistical Analysis

Data were analyzed using SPSS (v. 17.0) statistical software package (SPSS Inc., Chicago, IL, USA). Variables were examined for outliers and extreme values by means of box and normal quantile-quantile plots, and Shapiro–Wilk’s and Kolmogorov–Smirnov’s tests. When a normal distribution could not be accepted, variable transformations (square, square root, logarithmic, reciprocal of square root or reciprocal transformations) were reviewed and, if the normality could not be reached, (as for educational level and MMSE) nonparametric tests were used. One-way analysis of variance (ANOVA) was used to evaluate the differences between groups (AD vs. HS) in age, educational level, BMI and MMSE. Chi-square test was used to assess differences between groups in sex, blood group, comorbidity and drug intake. Group differences (HS vs. AD) were evaluated by uni- and multivariate analysis of covariance (ANCOVA), using age, sex, educational level, BMI, blood group, comorbidity and drug therapy as covariates. The assumption of the equality of variance was assessed by means of Levene’s test. Finally, correlation analysis was performed in each group by Pearson’s correlation coefficient (r) for normally distributed and Spearman rank correlation coefficient (rs) for not normally distributed variables, using Bonferroni’s correction for multiple comparisons. Correlation analysis was used to measure the strength and direction of association between IgG, IgM or IgA levels and age, sex, BMI, blood group, educational level, MMSE (as a measure of cognitive decline severity), comorbidity and drugs.

## 3. Results

Clinical and demographic characteristics of the subjects enrolled in the study are reported in Table 1. There were no significant differences between groups, excluding the higher educational level in HS.

Multivariate ANCOVA, including age, gender, educational level, BMI, blood group, comorbidity and drug therapy as covariates, showed a statistically significant difference (F = 13.566; df = 3.40; *p* < 0.001; partial η2 = 0.504) between groups (HS vs. AD). The results of univariate ANCOVA, as well as the serum levels of anti-Gal IgG, IgM and IgA are reported in Table 2. 

**Figure 1:**
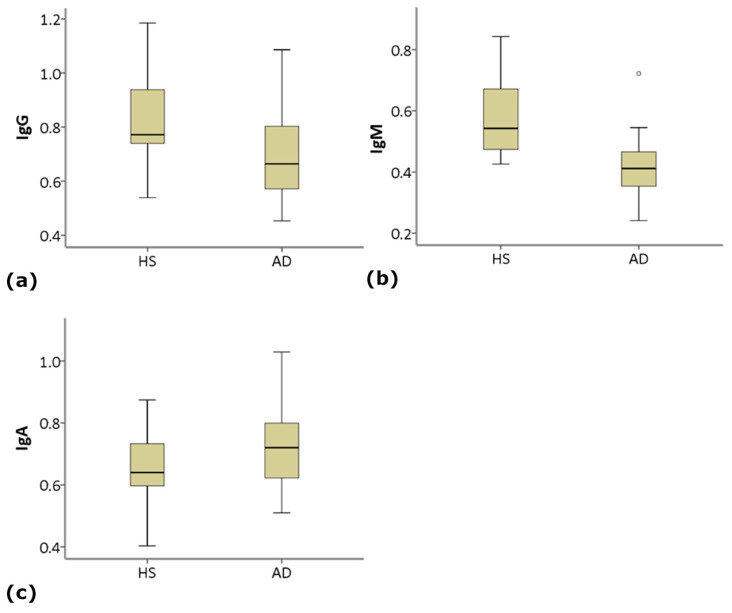
Serum levels (O.D. 450 nm) of anti-Gal IgG (**a**), IgM (**b**) and IgA (**c**) in the study groups. Box-plots show median (horizontal line in the box), 25th and 75th percentiles (edges of box), maximum and minimum values (whiskers) and outliers (°) of Ig levels in Alzheimer’s disease (AD) and healthy subjects (HS) groups (see Table 2 for statistical details).

IgG and IgM levels result significantly decreased, and IgA significantly increased, in AD patients compared to HS (Table 2; Figure 1).

**Figure 2:**
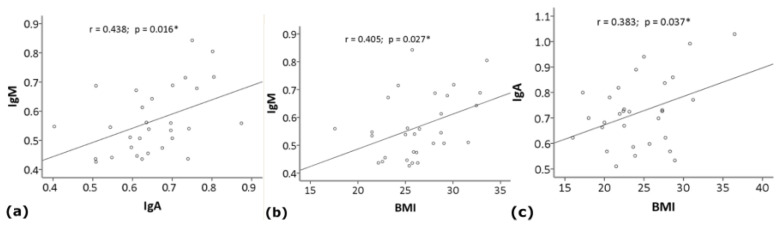
Scatter plots showing the correlation between serum levels (O.D. 450 nm) of (**a**) anti-Gal IgM and IgA (y = 0.49x + 0.25; R^2^ = 0.192) or (**b**) BMI (y = 0.01x + 0.24; R^2^ = 0.164) in healthy subjects, and (**c**) IgA and BMI (y = 0.01x + 0.45; R^2^ = 0.147) in Alzheimer’s disease patients. *, not significant after Bonferroni’s correction.

In the HS group, the analysis showed a weak positive correlation between IgM and IgA (r = 0.438; *p* = 0.016) or BMI (r = 0.405; *p* = 0.027), not significant after Bonferroni’s correction (Figure 2). In the AD group, the analysis showed a weak positive correlation between IgA and BMI (r = 0.383; *p* = 0.037), not significant after Bonferroni’s correction (Figure 2), and a trend toward a positive correlation between IgG and IgM (r = 0.351; *p* = 0.057).

No significant relationship was found between Ig levels and age, sex, blood group, educational level, MMSE, comorbidity and drugs.

## 4. Discussion

This study demonstrated that anti-Gal IgG and IgM were significantly decreased, and IgA significantly increased in AD patients compared to HS. No significant relationship was found between Ig levels and age, sex, blood group, severity of disease as assessed by MMSE, level of education, co-morbidity and drugs in AD patients, except for a weak positive correlation between IgA and BMI. To our knowledge, this is the first study that has investigated the serum levels of anti-Gal antibodies in AD and, therefore, comparison studies are lacking. However, circulating anti-Gal IgG results reduced compared with those of age/gender-matched HS in patients with MS [15], an autoimmune, inflammatory and neurodegenerative disease [27]. The authors speculated that a modified gut microbiota, specifically characterized by low α-Gal-producing microorganisms, can modify the inflammation homeostasis of individuals genetically predisposed, increasing the risk of developing MS [15]. The finding that gut microbiota of MS patients is characterized by a significant decrease in microorganisms expressing the GGTA1 gene (which codes for the α-Gal epitope) supports this hypothesis [16]. In patients with inflammatory bowel diseases, such as Crohn’s disease and ulcerative colitis, anti-Gal specific IgA resulted in higher concentrations than in HS, while total IgM resulted significantly decreased [28]. The authors speculated that the increase in IgA was linked to the presence of a damaged intestinal barrier exposing the subject’s immune system to enteric bacteria presenting α-Gal. The reduction of total IgM was interpreted as a switching of the antibody class [28].

Although anti-Gal antibodies do not meet the definition of natural antibodies (i.e., antibodies present in a non-immunised organism from birth), many authors place them in this category given the numerous features in common [8,9,10,14]. IgM and IgG are the most widely described classes of natural antibodies since they are implicated in many infectious diseases and pathologies, such as neurological disorders, cancer, diabetes and cardiovascular diseases. Interestingly, lower levels of natural antibodies are generally negatively correlated with disease onset and progress, whereas high levels often correlate with protection or absence of disease [9,14]. An example is represented by the IgG antibodies against amyloid peptides that were reported to be reduced in AD patients compared to age-matched HS [29] and to decrease with normal ageing and advancing AD, especially those binding to assemblies of amyloidogenic peptides [30]. The literature available on IgA is scant in contrast to IgM and IgG natural antibodies. IgA functions as a potent pro-inflammatory agent, being a rapid activator of neutrophils and it is crucial in the first line of defence against pathogens. In particular, they are considered homeostatic immunoglobulins that neutralize microbiota and food antigens to prevent interactions with the host [9,31].

We speculated that the simultaneous decreases in anti-Gal IgM and IgG and the increase in anti-Gal IgA we observed in AD patients might reflect the class switching of anti-α-Gal B cells from the production of “natural” IgM antibodies to an IgA-mediated adaptive response. Significant increases in serum IgA levels were observed in AD patients in comparison with HS [32,33,34]. An example is the IgA antibodies directed against the N-methyl-d-Aspartate receptor (NMDAR), detected in 10% of AD patients as opposed to 2.8% of HS [35]. The NMDAR NR1 subunit was found to carry glycostructures recognized by antibodies raised against the Fuc alpha 1-2Gal epitope [36], a common constituent of A, B and O blood groups. Noticeably, most anti-B antibodies in the sera of A and O blood group carriers are cross-reactive anti-alpha-Gal antibodies [37]. It is also of note that IgM and IgA-enriched Ig preparations incited the production of higher levels of TNF-α and NO by primary rat microglial cells in vitro, compared with IgG [38], which highlights the possible role of IgA-mediated responses in the microglial activation and neuroinflammation associated with AD. The phlogosis of lymphoid tissue associated with abdominal fat, promoted by excess caloric intake, and/or with a gut microbial dysbiosis, might alter the normal balance between tolerance and reaction to ubiquitary antigens in the gut-associated lymphoid tissue (GALT) favouring the sensitization of α-Gal-specific T helper 2 cells. The positive correlation that we observed between IgA and BMI and the encouraging results of therapeutic approaches aimed at controlling metabolic imbalance and inflammation for halting the progression of AD [39] are noteworthy in this regard. B cells activated in the GALT may home into the marginal zone of the spleen [40], wherefrom IgA produced by GALT plasma cells in response to commensal or pathogenic bacteria may enter the blood and permeate the BBB, in the presence of phlogosis.

The mounting of such an adaptive response to microbial α-Gal epitopes in the GALT and the concomitant permeabilization of the BBB to anti-Gal IgA antibodies may damage cells and tissues in the central nervous system as a result of antibody- and complement-mediated responses to: (a) formerly tolerated α-Gal epitopes also expressed by the brain microbiome [20,21]; (b) self-antigens containing identical or similar oligosaccharide moieties linked to autologous peptides, on the base of epitopic mimicry or epitopic spreading.

Gut microbial dysbiosis, frequently found in patients suffering from neurodegenerative diseases including AD [41,42,43,44], and which promotes the growth of a more aggressive intestinal bacterial population (Table 3) [45,46,47,48], may lead to the secretion of amyloid, lipopolysaccharides and other toxic substances (e.g., beta-N-methylamino-L-alanine, saxitoxin and anatoxin-alpha) [49,50], which alter both the gastrointestinal permeability and BBB favouring the passage of immunoglobulins, microorganisms and other molecules (toxic or not) into the brain [51].

The binding of IgA to α-Gal-like epitopes present on gray matter [18] and/or on the bacteria within neurons [21] may trigger complement-mediated cytolysis and a neuroinflammation process that, in turn, may provoke or exacerbate the amyloid cascade. This hypothesis is supported by the finding that bacteria belonging mainly to the Enterobacteriaceae family, including *Escherichia coli*, Pasteurellaceae genera, including *Haemophilus influenzae*, and Lactobacillaceae family bearing the GGTA1 gene [20] have been found post-mortem in the brain of AD and control subjects and all contribute to the brain microbiome [21]. The observation that senile plaques and neurofibrillary tangles are diffusely stained by using lectins obtained from *Griffonia simplicifolia* and *Amaranthus leucocarpus*, which are specific to α-Gal-like epitopes [19,52], also supports our hypothesis. Further to this, corpora amylacea and spherical deposits, frequently found in the brain of demented patients, also react positively when stained by lectins. Corpora amylacea have long been assumed to be a hallmark of normal brain ageing, however, their role appears to be similar to that of senile plaques and neurofibrillary tangles in the extent of cognitive dysfunction of individuals with dementia [19].

We acknowledge some limitations of our study due to its small sample sizes and the observational nature of the results. However, this novel observation opens up new potential research questions and may be a basis for establishing a new etiological factor for AD. In particular, our findings offer support to speculations linking α-Gal, gut microbiota, neuroinflammation and AD. A further study with a larger sample size and a longitudinal design is needed to examine the potential predictive value of anti-Gal antibodies as a biomarker of AD development and progression.

## 5. Conclusions

This study, the first to investigate the serum titers of anti-Gal antibodies in AD, showed lower IgG and IgM and higher IgA levels in AD patients compared to healthy subjects. These results suggest a possible role of anti-Gal immunoglobulins in the pathogenesis of AD and support the theory of the association between host microbiota, neuroinflammation and dementia. If confirmed in a longitudinal study with a larger number of participants, these findings could open new scenarios in the knowledge of AD etiopathogenesis and the research for new biomarkers and therapeutic strategies.

## Figures and Tables

**Table 1:** Demographic and clinical variables of study groups.

Variables	AD (N. 30)	HS (N. 30)	F (1,59)/X^2 #^	*p*
Females/males (N.)	19/11	15/15	1.086	0.297
Age (mean ± SD, y) (range, y)	83.77 ± 5.89 (70–96)	80.83 ± 6.04 (70–93)	3.631	0.062
Education level (mean + SD, y)	9.10 ± 5.27	11.77 ± 4.19	4.708	0.034
BMI (mean ± SD, kg/m^2^)	24.37 ± 4.56	26.37 ± 3.70	3.475	0.067
MMSE (score)	17.88 ± 6.85	30.07 ± 1.31	91.632	<0.001
Blood group (N; %)				
0	13; 43.3%	14; 46.6%	0.067	0.795
A	13; 43.3%	8; 26.6%	1.832	0.176
B	3; 10%	6; 20%	1.176	0.278
AB	1; 3.3%	2; 6.6%	0.351	0.554
Medical history (N; %)				
Smoke *	3; 10%	3; 10%	0.000	1.000
Dyslipidemia	11; 36.6%	12; 40%	0.071	0.791
Diabetes	8; 26.6%	6; 20%	0.373	0.542
Hypertension	17; 56.6%	19; 63.3 %	0.278	0.598
Myocardial infarction	3; 10%	3; 10%	0.000	1.000
TIA/Stroke	3; 10%	1; 3.33%	1.071	0.301
Drugs (N; %)				
Antihypertensive	17; 56.6%	18; 60%	0.069	0.793
Lipid-lowering	10; 30%	11; 36.6%	0.073	0.787
Hypoglycemic	8; 26.6%	6; 20%	0.373	0.542
Antiacid	12; 40%	11; 36.6%	0.071	0.791
Antiplatelet	13; 43.3%	12; 40%	0.069	0.793
Anti-inflammatory	3; 10%	3; 10%	0.000	1.000

*, current smoker; AD, Alzheimer’s disease; HS, healthy subjects; BMI, Body mass index; MMSE, Mini Mental State Examination; TIA, transient ischemic attack. ^#^, As described in the Methods section, F pertains to evaluation of age, schooling, BMI, MMSE, while X^2^ applies to all other parameters.

**Table 2:** Serum levels (mean ± standard deviation) of anti-Gal immunoglobulins in Alzheimer’s disease (AD) and healthy subjects (HS) groups and results of univariate ANCOVA (see statistical analysis section for details).

Ig (O.D. 450 nm)	AD	HS	Partial η2	F	df	*p*
IgG	0.696 ± 0.18	0.837 ± 0.16	0.121	5.784	(1, 42)	0.021
IgA	0.721 ± 0.13	0.652 ± 0.13	0.119	5.661	(1, 42)	0.022
IgM	0.413 ± 0.98	0.56 ± 0.11	0.419	30.313	(1, 42)	<0.001

**Table 3:** Variations in the intestinal bacterial population characterizing the gut microbiota dysbiosis in the presence of AD. Trends can be defined from data collection available in the literature.

Micro-Organisms	Increasing	Decreasing	References
Cyanobacteria	X		[42,50]
*Chlamydia pneumoniae*	X		[42,43,45]
*Borrelia burgdorferi*	X		[43,45]
*Escherichia coli*	X		[43,44,45,46,47]
*Shigella*	X		[43,44,45,46,47]
*Enterococcus*		X	[41,43,44,46,47]
*Blautia glucerasea/producta*	X		[41,48]
*Clostridium perfringens/saccharoliticum*		X	[41,44,45,47]
Gemellaceae (*Gemella*)	X		[41,48]
Mogibacteriaceae		X	[41,48]
Veillonellaceae (*Dialister*)		X	[41]
Tissirellaceae		X	[41,48]
Bifidobacteriaceae (*Bifidobacterium*)		X	[41]
Bacterodaceae (*Bacteroides*)	X		[41,44,45,46,47]
*Akkermansia*	X		[46]
*Bacillus subtilis*	X		[46,47]
*Klebsiella pneumonia*	X		[44,46]
*Mycobacterium* spp.	X		[46]
*Staphylococcus aureus*	X		[44,46]
*Streptococcus* spp.	X		[46]
Fusobacteriaceae		X	[47]
Prevotellaceae	X		[47]

## Data Availability

The datasets used and analyzed during the current study are available from the corresponding author on reasonable request.
